# The school as an arena for mental health work: exploring the perspectives of frontline professionals on mental health work in Norwegian schools

**DOI:** 10.3389/fpubh.2024.1454280

**Published:** 2024-10-10

**Authors:** Anita Berg, Lily Appoh, Kristin B. Ørjasæter

**Affiliations:** Faculty of Nursing and Health Sciences, Nord University, Namsos, Norway

**Keywords:** adolescents, children, focus groups, mental health work, roles, school professionals, sustainability, universal health promotion

## Abstract

**Introduction:**

Children and adolescents are increasingly facing mental health problems. Schools play a crucial role in promoting mental health, as they provide a unique setting where children interact with adults outside their homes.

**Methods:**

This paper explores mental health work in Norwegian schools from the perspective of frontline professionals namely, class teachers, social workers, and public health nurses. We conducted four focus group interviews involving 22 of these professionals from nine primary and secondary schools.

**Results:**

The school professionals view mental health work as an integral part of the school's mission and associate their work with promoting mental wellbeing, strengthening self-esteem, and building resilience among pupils. The professionals noted an expansion in their roles and an increase in expectations to undertake mental health work, even though it is not formally part of their job descriptions. Additionally, they play varied and complementary roles in supporting the pupils' mental health daily. As school professionals, they strive to balance universal health promotion with providing individualized mental health care.

**Discussion:**

These results call for coordinated efforts and interdisciplinary collaboration within the school and discussion regarding the school's role in mental health care for children and adolescents.

## 1 Introduction

The rising need for mental health care in schools highlights the essential services provided by school professionals (2). Mental health problems are becoming increasingly common among young people, posing a significant risk to their current wellbeing and future potential (3). The statistics are alarming, with adolescents experiencing mental health problems as the most prevalent non-communicable illness (4). Globally, one in seven youths aged 10–19 years suffers from mental illness (5). In Norway, mental illness prevalence varies by gender and age. About 7% of children aged 4–14 have a mental illness. Among adolescents, aged 13–16, the prevalence is around 8% for boys and 23% for girls (6).

The WHO advocates for schools to hold a central role in promoting pupils' health and wellbeing and has deemed initiatives like health-promoting schools and a whole-school approach as crucial (5). Schools are uniquely positioned to undertake health promotion (7, 8) and provide mental health services, given the considerable time children spend in schools under adult supervision (9). Proactively addressing mental health issues in educational settings can help to reduce the stigma associated with mental health challenges (10). Additionally, schools are well-suited to engage in health promotion, prevention, and remediation efforts (11). Furthermore, they can play a key role in identifying mental health problems (12, 13), preventing their escalation, and providing mental health services to support young people facing challenges (12, 14). While Norwegian authorities acknowledge the vital role schools play in children and adolescents' mental health (1), Norway still trails other Nordic countries in prioritizing school mental health initiatives.

Recent studies underscore the importance of cross-professional boundaries, where school professionals and collaborative partners from other sectors e.g., the healthcare and the social services must engage in multi-professional teamwork to achieve successful outcomes (7, 15). According to Dimitropoulos et al. (16), gaps remain in understanding school professionals' perspectives on their roles and contributions to mental health. While an increasing number of studies focus on the roles, experiences, and understanding of individual school professionals, few have addressed the whole-school perspective on school mental health work, which encompasses multiple professions within the same study context. This paper seeks to explore the perspectives of frontline professionals in Norwegian schools—class teachers, public health nurses, and social workers—on mental health work in school settings and their self-perceived roles, drawing upon their reflections from daily experiences working in Norwegian schools.

### 1.1 School professionals' roles and contributions in school mental health work

In our study, the focal school professionals are class teachers, public health nurses, and social workers, who predominantly serve in Norwegian schools today. This section delineates their qualifications, statutory positions, and roles in school mental health work.

### 1.2 Class teachers

The primary role of teachers is to educate children through classroom instruction [(17), §8-2]. Class teachers (CTs) play a pivotal role in creating a secure learning environment and managing practical, administrative, and social pedagogical tasks, including liaising with pupils' families (18). Their extensive interaction with pupils positions them well to identify mental health problems (16) and the teacher–pupil relationship is crucial for pupils' mental wellbeing (19). Teachers acknowledge their role in addressing pupils' mental health and advocate for increased information and support through collaborative efforts (12, 20). Research highlights teachers' unique position to identify mental health problems, while they emphasize their primary role as educators, not psychologists. They see their main collaborative role as identifying and observing problems, leaving follow-up to mental health professionals (20). Despite their desire for collaboration, challenges such as limited capacity, lack of mental health competence, and unclear roles hinder their efforts in supporting pupil mental wellbeing (8, 21).

### 1.3 Public health nurses

Public health nurses (PHN) in Norway are authorized nurses with a postgraduate education in public health nursing [(22), §3]. They have a particular responsibility for health promotion and disease prevention for children and adolescents up to age 20, along with their families (15). PHNs do not provide treatment but facilitate referrals for follow-up (6). They often oversee the school health service (SHS), a mandatory low-threshold service that should be easily available for pupils (15). Since 2018, the SHS has been a legally required interdisciplinary service [(22), §3]. However, PHNs are still most often the only professionals in the service (15, 23). A PHN is employed by the municipality and may serve multiple schools (13).

Prior studies indicate that a mere 14% of Norwegian schools provide daily PHN access (23), and over half of PHNs' time is spent on mental health problems (14). PHNs encounter several obstacles in promoting pupil mental health, including limited time, inconvenient SHS office locations, and a focus on “firefighting” mental health crises rather than on health promotion and universal primary prevention (45). The effectiveness of their work and interprofessional collaboration is shaped by regulatory frameworks, school governance, leadership, and the proactive engagement of teachers (15). To prioritize mental health work, PHNs advocate for full-time positions in every school and better integration with school teams (24).

### 1.4 School social workers

School social workers (SSWs) are key partners in fulfilling the school's mission of educating resilient pupils who are prepared to navigate the complexities of wellbeing and life's challenges (25). However, their role in Norwegian schools remains unclear (26). Unlike teachers and PHNs, SSWs do not have statutory tasks within schools (27), nor do they operate under national standards or guidelines that shape their work in school settings (28). They are often employed as milieu therapists, holding informal and self-initiated roles (29). Holmøy et al. (30) emphasized the need to clarify responsibilities and formalize social workers' tasks in schools—specifically, which tasks should be addressed universally and which should target specific groups. Thus, SSWs are deeply involved in school mental health work (31), supporting not only vulnerable and struggling pupils but also the broader school community (25). SSWs are recognized as psychosocial experts within schools (32). Traditionally, they act as primary facilitators of communication between the school, home, and community, serving in various roles, such as therapists, mediators, and “garbage cans” (33).

## 2 Materials and methods

This study explores the perspectives of CTs, PHNs, and SSWs on mental health work in a Norwegian municipality. The study emerged from a national effort to enhance youth mental health and wellbeing through local knowledge-based interventions, coordinated by the National Program for Public Health Work in Norwegian municipalities during the period 2021–2027 (6).

### 2.1 Recruitment and participants

We employed purposive sampling, specifically maximum variation sampling, to select participants who could provide valuable and relevant data for our research objectives and offer insights form different perspectives (34). In the Local Program for Public Health, a public health coordinator served as the gatekeeper. This municipal coordinator's role included ensuring diverse representation from various basic schools within the municipality, considering factors like size, location, and educational levels. Additionally, the gatekeeper actively recruited school professionals who interact daily with pupils and their parents, encompassing both compulsory education from primary school (ages 6–13) to lower secondary school (ages 13–16). The main criteria for inclusion in the study were (a) being employed as a CT, PHN, or SSW in basic schools for a minimum of 2 years and (b) having regular contact with pupils (ages 6–16) and their parents, in primary and/or secondary schools.

Initially, all PHN and SSW employed at the target schools were invited to participate in the study due to their limited number. Due to the greater number of CTs at these schools, principals selected one or two teachers from both the primary and secondary levels. To maintain impartiality and prevent a single perspective from prevailing, professionals involved in the working group for the Local Program for Public Health were excluded.

The total sample included 22 professionals from nine schools in one Norwegian municipality. The sample consisted of seven CTs in primary school, six CTs in secondary school, five PHNs, and four SSWs. The focus groups balanced homogeneity and diversity by being homogeneous by profession but mixed between schools to capture varied experiences. Most participants had extensive work experience, except for a few with < 5 years. Participants were aged 25–55, with only two men among the 22.

### 2.2 Conducting focus groups

We held four focus groups consisting of four to seven participants. Focus groups are effective in capturing the insights, understandings, and values of participants, which can guide the development of programs, policies, or services. Additionally, the focus group setting encourages individuals to share their thoughts freely and voice reflections not previously vocalized, enriching the dialogue (35). In our study, focus groups were selected over individual interviews to uncover diverse perspectives, stimulate dialogue, and leverage the synergistic effect, allowing participants to build on each other ideas, resulting in richer and more creative insights.

The focus group sessions were conducted in person in the spring of 2019, with the first and third authors serving as moderator and assistant moderator, respectively. Each session lasted between 90 and 120 min and included key open-ended questions formulated specifically for our study. These questions included: “How do you understand the concept of mental health?,” “What do you consider central to promoting mental health in children and adolescents?,” and “In what way do you collaborate with other professionals to promote mental health among children and adolescents?” The focus groups were audio recorded and a professional transcriber transcribed all interviews verbatim.

### 2.3 Data analysis

In our data analysis, we collaboratively engaged in a reflexive thematic analysis (36). This methodological approach aims to maintain a systematic, phase-based structure while ensuring that active, reflexive, and recursive processes evolve through data interpretation (36). By adopting a “bottom-up analysis”, where themes emerge from the data itself rather than pre-existing theories, we aimed to provide a coherent and compelling interpretation of the data. We carried out the analysis in six phases (37):

Phase 1: We read the transcribed interviews multiple times to become familiar with the material. During our readings, we jotted down and discussed thoughts and reflections.

Phase 2: The transcribed interviews were broken down into smaller parts. For each interview, we generated initial codes, mind maps, and tables.

Phase 3: This phase marked a shift in focus to interpreting the aggregated meanings and meaningfulness across the dataset. Potential main and sub-themes were developed.

Phase 4: We performed a recursive review, refining our main and sub-themes to ensure they reflected the data. This phase culminated in a clear comprehension of our themes, their interrelations, and the overarching narrative they presented.

Phase 5: We defined and reviewed the themes, paying special attention to the naming of the main and sub-themes to ensure a coherent interpretation of the data.

Phase 6: In this phase we wrote, reflected on, revised and condensed the analytic text into the final presentation of the results.

### 2.4 Ethical considerations

The study adhered to Norwegian ethical research standards, receiving approval from the Norwegian Agency for Shared Services in Education and Research (Sikt/2019, no. 987669). Participation was voluntary and confidential, with participants free to withdraw without explanation. Informed consent was secured, and anonymity was maintained through identification numbers and abbreviations corresponding to their professions: public health nurses (PHN), school social workers (SSW), and class teachers in primary school (CTPS) or secondary school (CTSS).

## 3 Results

In exploring the school professionals' perspectives on mental health work, including their self-perceived roles and practices, the analysis identifies three main themes: (1) mental health work as a positive approach in schools, (2) school professionals as mental health workers, and (3) the tensions between policy and practice in school-based mental health work.

### 3.1 Mental health work as a positive approach in schools

The participants articulate an overall positive stance on mental health, regardless of professional backgrounds. This positive focus manifests in three interrelated understandings of mental health work, the three sub-themes: promoting mental wellness, strengthening self-esteem, and building resilience.

Firstly, participants emphasize the pivotal role of mental health work in promoting a sense of mental wellbeing among pupils. They perceive their responsibilities as assisting pupils toward inner peace. As SSW1 eloquently put it, they aim for pupils to “rest in themselves as human beings”. Additionally, CTSS2 emphasized the importance of professionals ensuring “that the kids feel fine”. The participants' understanding of mental wellness is firmly anchored in the present moment, considering the wellbeing of pupils within the context of their everyday lives.

Secondly, mental health work in schools actively seeks to foster robust self-esteem and self-worth among pupils. Participants believe that robust self-esteem does not originate on its own. Therefore, they stress the importance of focusing on pupils' strengths and potential rather than their shortcomings. They advocate for bolstering pupils' self-esteem by supporting their unique value as human beings, independent of their performance or accomplishments:

We must make them realize that they are good enough as they are... and if [the pupils] don't fix everything right now, that is also okay. There are so many other things [they] are good at (CTP4).

Participants acknowledge their pivotal role in empowering pupils to develop self-worth. As emphasized by SSW1, “we must enable them to develop as self-confident humans”. For the participants, it is essential to nurture pupils' self-esteem based on their value as human beings, not solely on their achievements, as part of their approach to mental health work.

Finally, mental health work focuses on cultivating resilience. Participants advocate the development of resilience and robustness, equipping pupils to navigate and grow through adverse situations. Nevertheless, they voice concerns over the current generation's limited understanding and training in managing adversities as part of normal life: “A lot of pillows are sewn under the arms of today's youth. They have not learned to cope with adversity themselves” (SSW3). The participants underscore the importance of pupils' ability to recognize, understand, and regulate their emotions as crucial to resilience and improved mental health. They highlight the significance of emotional coping strategies for resilience, stating “First, they should learn to recognize their feelings, understand them, and acknowledge them. Second, they should learn how to deal with their negative emotions” (PHN2).

### 3.2 School professionals as mental health workers

All participants actively engage in mental health work in schools, recognizing it as key to their roles. This engagement involves, as described in theme one, promoting mental wellness, strengthening self-esteem, and building resilience among pupils. Despite the absence of explicit definitions in their job descriptions, they deem this work as essential to complying with their role expectations and addressing pupils' needs. The upcoming sections detail the perceived roles of CTs, PHNs, and SSWs in school mental health.

Class teachers, by engaging consistently in the daily school lives of their pupils, play a pivotal role as relationship builders, daily carers, and reality orientators. One CT expressed, “As a class teacher, I believe it is my responsibility to foster positive relationships with everyone in the class” (CTP3). Class teachers adapt their roles to align with the developmental phases of their pupils, providing comfort and managing conflicts in primary school while promoting stability and supporting pupils through severe life challenges in secondary school. From their perspectives, pupils often harbor numerous worries, ranging from mundane to serious, all requiring adult guidance: “We are the frontline professionals, being the adults standing by their side in their daily life” (CTPS2). As key contributors to pupils' mental health, CTs actively engage in discussions about the expectations and limitations of their competence in mental health work.

When it comes to mental health... it is expected that I should be quite competent. This foremost includes expectations from the parents but also the municipality. The society or system expects us to be enormously skilled. Of course, we are skilled, but we have our limits. (CTSS3)

The divide between CTs' formal duties, the needs they perceive, and the observed expectations underscores that their responsibility has limits.

Public health nurses perceive their roles as disseminators of universal knowledge and responders to individual emergencies. Within the basic school setting, they focus on universal health promotion and disease prevention. Their duties encompass sharing knowledge on healthy lifestyles with both parents and pupils, performing health assessments at given checkpoints, and offering accessible low-threshold counseling services.

In their daily practice, they serve as disseminators of knowledge and emergency responders, addressing specific and severe health problems faced by pupils. They navigate the dichotomy between their mission of broad health promotion and the increased need for personalized follow-up. One PHN articulated this conflict, stating, “The national guidelines require us to focus on universal prevention, but in practice, we are doing too much firefighting on mental health problems” (PHN1). This tension is particularly pronounced in secondary schools, where the demand for mental health aid challenges their foundational objectives.

The PHNs perceive that the inefficient organization of their services within the municipality hinders the effective use of their competence due to their exclusion from mental health work in the schools. As non-permanent fixtures in the schools, they experience their presence as transient visitors, with one PHN sharing: “I feel like I'm flying in like a UFO, into the [public health nurse] office and … [when time is up] out again … I feel we do not get the opportunity to connect to the schools” (PHN1). As isolated professionals, PHNs manage predefined duties across multiple schools, with limited opportunities to prioritize tasks and collaborate with colleagues.

In contrast to PHNs' strict, predefined roles and timelines for their work, school social workers hold positions as milieu therapists, maintaining a visible and approachable presence in school settings that facilitates low-threshold interactions. SSWs embody the roles of low-threshold interlocutors and bridge-builders. As one SSW noted, “I have such a freer role”, highlighting the flexibility to adapt their tasks to the immediate needs of the pupils. Another SSW reflected on what this autonomy entailed in terms of mental health work in school: “The social workers act perhaps more directly towards the pupils … driven by their needs” (SSW3). SSWs proactively engage with pupils based on their requirements, which involves a 2-fold mission. First, they empower pupils to self-navigate the complexities of “growing up in contemporary society”, emphasizing self-acceptance and confidence-building. They consider personal development to be equally critical as academic achievement: “If we do not make them aware of their inbuilt strengths before focusing on learning, we will fail enormously” (SSW1).

Aside from their proactive health-promoting mission, SSWs also assist pupils with severe mental health problems who need specialized care. This reactive mission, not originally in their job description, emerged due to long waiting times for child welfare and child and adolescent psychiatry. As one SSW stated:

We are the ones meeting these young people every day … I know very well that it takes 3–5 months from referral to the first appointment with specialists. I cannot be at peace when I see a deterioration from day to day. (SSW1)

Consequently, SSWs assume their reactive role of decoding pupils' problems into the terminology used by external services, drawing on insights from teachers, parents, and the pupils themselves. This translation helps decision-makers expedite the pupil's progression through the system. Additionally, the SSWs reactive role entailed acting as bridge-builders among schools, families, and external services, fostering constructive communication and enhancing collaboration to help pupils with, or at risk of developing severe mental health problems.

### 3.3 Tensions between policy and practice in school mental health work

In the context of school mental health work, participants experience tensions arising from the interplay between policy expectations and the practical realities they encounter daily. This analysis identifies three key sub-themes: the expanded role of schools in addressing mental health problems, finding the balance between prevention and crisis management, and the imperative for low-threshold interdisciplinary collaboration.

Participants recognize an expanded mandate for schools in the realm of mental health work, attributing the expansion to two interrelated changes: Firstly, an increased need for mental health support for children and adolescents due to insecurity in the parental role and mental health problems in the families. As articulated by a primary CT: “There are more and more kids who are having problems. It can be their own mental health, but it can be their mother or father, or illness in the family” (CTP3). Secondly, an expanded role relates to health service capacity challenges in both the municipal support system and the specialist health service. Coupled with the policy of providing mental health service at the lowest executive level, this creates problems in schools, as they are tasked with supporting at-risk groups and pupils with mental health problems without the resources to perform mental health aid on a large scale or provide follow-ups with pupils in need of specialized care. One SSW expresses the predicament:

When I am in contact with almost one-third of all the secondary school pupils and their parents regarding problems … it is not possible for me to reach out to 100 pupils and 200 parents during my working hours. It is impossible. (SSW1)

Participants trained in mental health care notice a change in the complexity and severity of pupils' needs, stating: “It is a lot more complex today and a lot of different diagnoses … We should not be the ones treating depressions and helping young people who are suicidal. That's not our job” (PHN1). The complexity and severity of the pupils' need puts pressure on the school professionals to act and address what they perceive as immediate mental health crises. They experience lack of formal recognition of these challenges, and their attempts to refer the pupils often fail due to increase in pressure and queues at the municipal and specialist mental healthcare services.

Participants actively navigate universal health promotion and targeted individual interventions. They underscore the significance of preventive strategies within schools and championing universal prevention as a crucial approach to preventing or minimizing the occurrence of problems, benefiting both individuals and society. Despite great confidence in health promotion and disease prevention, the participants find that their daily efforts predominantly revolve around crisis management, colloquially termed “firefighting”. This mismatch between the terrain and map is exemplified by two PHNs:

PHN2: We get constant reminders from our leader, who is concerned with us working universally. Our job is to work with primary prevention … and we are quick to refer pupils [to specialized health care]. But we also have a lot of pupils ourselves, because there is no one else … in the municipality to take care of them.

PHN3: Psychiatrists or the child protective services refer them [back here] to us. … We are the ones to offer them treatment, and that is wrong … that we are the ones who must deal with the lack of follow up from the other services.

Participants acknowledge the unsustainable nature of current practices in addressing children and adolescents' mental health if they are to succeed in their primary task of universal disease prevention and health promotion.

Finally, participants recognize the need to develop low-threshold interdisciplinary collaboration. They posit that effective support for pupils requires collaborative effort within the school and with external partners, stating, “Proper efforts towards pupils in the schools, we cannot do it alone. We must work as a team, together with the school and other professionals, not to forget the parents and the child themselves of course” (PHN1). The pursuit of interdisciplinary collaboration presents a valuable opportunity to draw a more complete picture of the issues affecting the pupils' mental health and to intervene together in a low-threshold manner:

I think we have come a long way if we manage to think interdisciplinarity in school. If we are provided with a sufficient number of professionals from different specialties, then we can work together, discuss and elaborate things from different angles. (CTSS2)

Participants note that merely increasing competence in schools is insufficient. They stress the necessity for work organization that capitalizes on interdisciplinary collaboration within schools and in coordination with other municipal services. Instead of assuming that a low-threshold service would involve extra costs and demand more professionals, they underline the importance of assessing the entire municipal workforce to determine if personnel could be used more effectively: “We have the competence in the municipality … It is there, but must be coordinated … I think there is a lot of expertise, but it is used so randomly” (SSW1). To fulfill the schools' role as a health-promoting arena, participants propose low-threshold interdisciplinary collaborations, both in the school setting and extending to external partnerships, to enhance pupils' mental health.

## 4 Discussion

Our study found that school professionals recognized mental health work as a fundamental and inherent aspect of their daily work and a necessity in fulfilling the school's mission. They experienced an increased need to strengthen mental health work in school due to pupils' needs, capacity issues in mental health care, and policy demands. Against this backdrop, our discussion will cover (1) the school's mission and the expanded role that school professionals have assumed, (2) the balance between mental health promotion and individual mental health follow-up, and (3) task distribution and collaboration within the school and with external partners to address the growing mental health problems among pupils.

For our participants, mental health work within schools involves enhancing mental health as a valuable resource, affirming pupils' mental wellness, and helping them become self-confident, robust, and resilient. This aligns with previous research (9, 38) and Norwegian policies (1). The study's participants recognized that facing adversity and challenges is a part of life and that they could guide pupils through such challenges. They viewed it as a natural aspect of their role as adults within the school to support students in navigating life's ups and downs, drawing upon their competence and experience. Additionally, they aimed to identify and assist those who need additional mental health care. The professionals in our study view their roles and missions in mental health work in relation to their primary responsibility, their unique competencies, and their positions held in the school system.

Teachers viewed themselves as the primary enablers of a health-promoting and positive learning environment, acting as daily carers for pupils and fostering strong relationships with them, their peers, and their families. This role aligns with findings from prior Norwegian studies (18, 19) highlighting the role of CTs in providing a safe psychosocial school atmosphere. Due to their close proximity to pupils' everyday activities and their ability to observe them over time, CTs are in a unique position to identify mental health problems. This aligns with Dimitropoulos et al.'s (16) concept of teachers “seeing the red flag first” by establishing and maintaining strong relationships with pupils. However, CTs acknowledge that it is PHNs and SSWs, rather than themselves, who possess the essential expertise to support pupils facing mental health problems.

PHNs experienced the clearest mandate regarding their mental health work and understood their role in providing health information, monitoring health, and following up with pupils facing specific and severe health challenges. Their work was grounded in explicit guidelines and predefined tasks. Due to the increased number of pupils experiencing mental health problems, they called for full-time positions in schools. This call was echoed in a recent review by Kaskoun and McCabe (24). However, the PHNs were clear that they should not be the ones providing mental health aid to pupils with severe mental illnesses. This finding supports the study of Dahl and Clancy (39), in which PHNs described themselves as generalists. As solitary practitioners who spend most of their time in their offices, the PHNs called for better interdisciplinary collaboration with other school professionals.

SSWs carried a unique responsibility for accessible, low-threshold work. They served as key discussion partners for CTs and PHNs, establishing connections among pupils, families, and external services. Despite lacking statutory tasks (27), our study acknowledged SSWs' active engagement in school mental health work (31). In their more flexible roles, SSWs often pulled strings for pupils facing severe mental health problems, emphasizing empowerment and self-confidence. Despite unclear role definitions, SSWs held key positions in addressing mental health needs and were recognized as psychosocial experts (32) promoting wellbeing throughout the school (25). Positioned beyond the confines of classrooms and PHN offices, they acted as vital connectors, facilitating cohesion and support across the school's educational network.

In essence, school professionals' focus on school mental health work was 2-fold: a present focus that involves promoting the pupils' wellbeing by facilitating a positive psychosocial school milieu and a future focus that involves equipping pupils with the skills needed to cope with adult life. The present and future foci are in line with the view of mental wellbeing as a necessity for effective learning and a nurturing school milieu as a resource in helping pupils to grow into confident, robust, and resilient adults (12, 40). Our findings show that school professionals emphasize collaboration, leveraging each other's expertise and skills in mental health work. They viewed this work as a joint project, with each professional contributing complementary knowledge, roles, and skills. This collaborative stance, which values disciplinary diversity and joint efforts, reinforces previous research by Borg and Pålshaugen (41) and Dimitropoulos et al. (16), highlighting interdisciplinary collaboration as the key to success in school mental health work. Interdisciplinary collaboration and joint commitment to promote pupils' mental health underpins the proposed whole-school approach (5). This may indicate that staff attitudes, values and possibilities to unite their effort are fundamental for enabling health promotion in schools. In our study of frontline professionals' perspectives on mental health work in schools, we found the same barriers as in prior studies of health promotion in schools.

A prominent finding of this study is the challenge posed by the rising incidence of mental health problems and illnesses among pupils, which strains the capacity of school professionals to focus on universal mental health promotion. Consequently, these professionals face shortages of time, resources, and opportunities for effective collaboration, which hinders their mission to use schools as platforms for mental health promotion. They often find themselves pulled toward providing mental health care that exceeds their competence, job description, and capacity. The increased number of pupils requiring specific attention for mental health follow-up, along with the severity of their problems, raises concerns about whether school professionals are adequately positioned, trained, and equipped with the necessary competence and capacity to meet these needs. Participants in our study report limited support from other services, with some feeling isolated due to the infrequent external services engagement, leading to a lack of firsthand observation of the pupils in question. They were concerned by the redirected responsibility from child and adolescent psychiatry services, citing insufficient time and competence to manage these duties. This aligns with Svensson and Warne's (42) findings, which revealed a heavy burden on teachers and a skepticism toward child and adolescent psychiatry services, which often operate on second-hand information and provide advice without direct pupil interaction. Shelemy et al. (43) also emphasized the need for enhanced communication and engagement with external services, especially child and adolescent mental health services.

Our findings underscore the urgent need for cross-sectoral dialogue to ensure a collaborative and well-resourced approach to the wellbeing of pupils. It is essential to critically evaluate the roles that school professionals together with external mental health services should hold to fulfill the schools mission in mental health work. A recent review by Zabek et al. (44) proposed maximizing the use of school mental health professionals e.g., through task shifting and by using their competence to supervise their non- health professional colleagues as well as developing and implementing comprehensive school mental health systems. This raises the question of whether the schools professionals should maintain their current roles and responsibilities, or if their roles, labor division and collaboration have to be redefined to align with the evolving needs of young people.

Investigating the experiences of similar countries in organizing and distributing responsibilities within the domain of school mental health services across diverse professional categories and care tiers could prove beneficial. In line with the Swedish researchers Svensson and Warne (42), who advocate for clearer organization and consensus in supporting pupils with mental health problems, our study underscores a national-level debate on sustainable mental health promotion and care for children and adolescents in Norway. This debate must transcend individual sectors to embrace a comprehensive approach to pupil mental health, extending past the dimensioning and task distribution within the current school organization's mental health work. Addressing the overall allocation of responsibilities between schools and mental health services is imperative.

## 5 Strengths and limitations

Given the scarcity of Norwegian research on school frontline professionals in general, this study enriches the field by incorporating the perspectives of three types of professionals who concurrently serve in the same municipality. The study involved a limited number of participants and only included professionals directly involved in following up with pupils and their parents regarding everyday school activities. Thus, due to the inclusion criteria, all the school professionals had recent experiences and inside knowledge of the school's inner ecosystem. Expanding the number of participants and including professionals at the management level would have strengthened the study's design in terms of capturing the whole-school perspective.

The data were collected in homogenous focus groups with participants from different schools. Mixing groups with unfamiliar participants across schools may have impacted the individual participants in sharing their personal experiences and conflicting views. However, the focus group methodology enriched the data with in-depth explanations and discussions due to the participants' eagerness to share their perspectives and experiences with each other across school contexts. Thus, the study makes no claim to representativeness; instead, it seeks to explore the perspectives of frontline professionals on mental health work in Norwegian schools. Contributing to the literature, this study enhances the body of knowledge by enabling comparisons of school mental health work across professions and borders.

## 6 Conclusion

The findings from our study demonstrate that school professionals regard mental health work as a crucial component of their roles, necessary for fulfilling their primary responsibility within the school. The school professionals noted an expansion in their roles and heightened expectations to undertake mental health tasks, influenced by pupils' needs and expectations from parents, health services in the municipalities, and specialized health care.

The primary challenge for school professionals lies in striking a balance between their main mission of universal health promotion and the provision of mental health follow-up to pupils with significant mental health problems. The results indicate weaknesses in current mental health services for Norwegian pupils in relation to the division of tasks and capacity. This study is a reminder to policy-makers not to overestimate the capacity of schools and underestimate the significance of interdisciplinary and cross-sectional mental health work. Further research is needed to explore the opportunities and barriers that affect school professionals' ability to fulfill schools' mission in mental health work for children and adolescents. Interdisciplinary and cross-sectional collaboration appear to be prominent pathways to pursue in both research and policy development.

## Data Availability

The datasets presented in this article are not readily available because the data is restricted by the consent given by the participants. Requests to access the datasets should be directed to anita.berg@nord.no.
